# Role of solute carrier transporters in the biodistribution and toxicity of chemotherapeutic drugs

**DOI:** 10.1016/j.pharmr.2026.100117

**Published:** 2026-01-16

**Authors:** Mike Boeckman, Thomas Drabison, Arthur Germakovski, Allison Warmuth, Bagdad Ahmed, Anika T. Chowdhury, Shuiying Hu, Jason A. Sprowl, Alex Sparreboom, Kevin M. Huang

**Affiliations:** 1 Division of Pharmaceutics and Pharmacology, College of Pharmacy & Comprehensive Cancer Center, The Ohio State University, Columbus, Ohio; 2 Department of Pharmaceutical Sciences, School of Pharmacy and Pharmaceutical Sciences, Division of Molecular Biosciences, University at Buffalo, Buffalo, New York

## Abstract

Our understanding of the solute carrier (SLC) family of transporters has greatly increased in recent years, especially in oncology, and a wealth of information is now available, indicating that certain SLC family members contribute to the cellular accumulation of small-molecule cancer drugs at sites of injury and to unwanted toxicity in normal tissues. The present review aimed to provide an overview of the toxic effects of commonly used chemotherapy drugs that are associated with SLC-mediated transport, how these associations have been derived, what ensuing intervention strategies have been explored, and how the investigation of these phenomena might change in the near future with the availability of increasingly sophisticated and innovative models and techniques. It is expected that this rapidly emerging field continues to contribute to filling our gaps in knowledge and will aid in the development of interventions aimed at preventing debilitating side effects of cancer drugs and improving the quality of life.

**Significance Statement:**

Toxicities associated with small-molecule chemotherapeutics can be debilitating or even life-threatening and pose a burden on the healthcare system. Improving our understanding of the initiating transporter-mediated mechanisms of these side effects is crucial to the development of preventative or treatment strategies.

## I Introduction

Longstanding clinical oncology strategies to address the treatment of cancer rely upon complex combinations of surgery, radiation therapy, and chemotherapy. Current treatment approaches can differ greatly based upon tumor type, disease stage, and patient comorbidities. However, small-molecule chemotherapeutics remain a cornerstone of treatment strategies for a large proportion of cancer patients in a neoadjuvant or adjuvant setting, owing to their clinical benefit. Cytotoxic drugs tend to be broadly active in highly proliferative cells, making them attractive choices across cancer contexts as a single agent or in combination with other modalities. Although effective against many malignant diseases, cytotoxic chemotherapy often causes debilitating adverse effects in patients. Indeed, toxic effects are a leading cause of cessation of therapy in the clinic,^1^ and projected toxic effects, even if likely to be considered low-grade, can dissuade patients from accepting or continuing treatment for their cancer.^2^ This has led to the recognition that low-grade adverse events can be important mediators of overall tolerability and quality of life.^1^ The injury caused by these agents can greatly complicate patient care and can lead to dose reduction or discontinuation of an otherwise effective therapy.^3^ This problem has largely not been improved with the advent of targeted therapies such as protein kinase inhibitors,^4^ immune checkpoint inhibitors, antibody-drug conjugates (ADCs), and chimeric antigen receptor T cells.^5^, ^6^, ^7^ Acute and chronic adverse events induced by agents remain an immense burden on patients and healthcare systems alike.^8^^,^^9^ In addition, the compounded effects on toxicity caused by changes in drug exposure associated with polypharmacy regimens and with end-stage organ dysfunction that interfere with rate-limiting absorption and elimination processes are particularly problematic in elderly patients with comorbidities. These toxicities can also become lifelong functional impairments after years or even decades of survivorship following the discontinuation of a successful therapy.

The thesis that cell-specific expression of particular xenobiotic transporters serves as a mechanism governing the uptake of chemotherapeutics to concentrate in healthy organs for a selective injury to targeted cells has been explored for many important oncology drugs. This is based on the assumption that (1) the accumulation of drugs over a threshold concentration in healthy tissues is a prerequisite for subsequent cellular injury that manifests as a clinically significant toxicity, (2) the drugs can “hitchhike” on uptake transporter proteins referred to as solute carriers (SLC) that have evolved to mediate cellular uptake of structurally similar endogenous or exogenous compounds; and (3) the dominant mechanism of uptake of these problem drugs is mediated by 1 or more identifiable SLCs. The SLC superfamily contains 65 gene families and collectively encodes 458 transmembrane transporter proteins that serve as evolutionarily conserved gatekeeps that govern the selective movement of substrates between the extracellular and intracellular environment.^10^ SLCs recognize not only a broad array of essential nutrients and signaling hormones but also an everincreasing number of xenobiotic compounds, including cancer therapeutics. These transporters facilitate the movement of solutes across biological membranes that are adenosine triphosphate (ATP)-independent but can demonstrate dependencies on sodium, pH, and substrate concentration gradient.^11^

Any discussion on transmembrane transport and drug accumulation would be incomplete without introducing ATP-binding cassette (ABC) transporters. ABC transporters utilize ATP to pump substrates out of cells, including chemotherapeutic drugs.^12^ The functional expression of ABC transporters is involved in several absorption, distribution, metabolism, excretion processes and has been identified to contribute to chemoresistance and drug-induced toxicity.^13^, ^14^, ^15^, ^16^, ^17^ ABC transporters are largely outside the scope of the current review, and therefore, it will not be discussed in detail. However, it is important to highlight that the net balance between drug uptake and drug efflux will govern intracellular drug levels and drug action.

Of particular interest within the SLC superfamily are the cationic and anionic transporters, since these are recognized to transport a wide array of xenobiotics, including small-molecule cancer drugs, across cell membranes.^18^, ^19^, ^20^ These transporters include the organic anion transporters (OATs), organic cation transporters (OCTs), organic anion transporting polypeptides (OATPs), and multidrug and toxin extrusion proteins (MATEs), which by virtue of their expression profiles in organs of elimination, have all been implicated as determinants in the pharmacokinetic profile of xenobiotics.^21^, ^22^, ^23^, ^24^ Thus, changes in the function of these transporters resulting from genetic predisposition or pharmacological interference may have important clinical consequences for drug efficacy and drug safety. In the precloning era, it was believed that most drugs would cross the cell membrane simply by passive diffusion, assuming a favorable concentration gradient and sufficient lipophilicity. However, it has been increasingly evident that transport by SLC transporters (alongside ABC transporters) may be the predominant mechanism by which drugs move across the cell membrane and distribute throughout the body.^25^, ^26^, ^27^ Accumulating evidence suggests that tissue-specific uptake of drugs by SLCs is crucial for the tissue distribution and elimination of these drugs and can be an initiating mechanism driving observed toxicity profiles.^28^^,^^29^ This thesis is consistent with the notion that many small-molecule cancer drugs are charged under physiological conditions and distribute in a tissue-specific manner that coincides with organs that are targets of injury.

Initial evidence that supported the existence of transporter-mediated processes occurring independently of passive diffusion was inferred as early as the 1960s from observations made with methotrexate, an antimetabolite, and the reduced folate carrier (RFC).^30^ In the precloning era, the functional deficiencies in cells lacking the RFC gene, an uptake transport mechanism, were identified as a potential mechanism of resistance to methotrexate in cell-based models of leukemia.^31^^,^^32^ In the 1990s, recombinant DNA technology was used to clone the *RFC1* gene, now known as SLC19A1, to further confirm the existence of a transporter-dependent mechanism.^33^ Taken together with emerging studies on efflux transporters, particularly within members of the family of ABC transporters, views have emerged that the extent to which drugs accumulate in cells is a net result of both uptake and efflux processes. Uptake mechanisms can be rate-limiting in this process and significantly contribute to saturable kinetics.^34^^,^^35^ The original studies with methotrexate and SLC19A1 have informed the way we consider the transport of other small-molecule chemotherapeutics and how interference of these mechanisms can affect the pharmacokinetic and pharmacodynamic profiles of many drugs.^32^ At present, there now exists a large knowledge base with evidence to support the notions that (1) SLCs expressed at sites of injury, including cancerous and healthy tissues, are often a prerequisite for chemotherapeutic agents to induce cytotoxic effects, (2) SLCs expressed in organs of elimination such as the liver and kidney can be rate-limiting for hepatic biotransformation and renal excretion, respectively, and (3) perturbations of SLC function can have direct implications on drug efficacy, drug safety, or the ability to maintain tissue homeostasis. The following provides a supporting perspective on the role of SLC transporters and their contribution to the biodistribution and toxicity of chemotherapeutic drugs. It is important to recognize that notable reviews on SLC and ABC transporters and the toxicity of chemotherapeutic drugs have been previously published and that the current review is not exhaustive due to the number of genes within this family.^24^^,^^27^^,^^28^^,^^34^^,^^36^, ^37^, ^38^ The following will first provide an overview of the recent advances in research models of SLC transporters, followed by the role of SLCs in the transport of chemotherapeutic drug families of platinum agents, nucleoside analogs, anthracyclines, Vinca alkaloids, taxanes, tyrosine kinase inhibitors, and, more recently, payloads of ADCs.

### A Novel research models

The direct uptake of radiolabeled or fluorescent substrates into genetically engineered cell-based models, or murine and human primary cells, is a foundational method in investigating the function of xenobiotic transporters. Recently, technological and methodological advances have revolutionized transporter research, enabling a more precise and efficient exploration of elucidating the interplay between transporters and their biological role in tissues. Such technologies include gain-of-function SLC libraries, containing codon-optimized cDNAs, as well as clustered regularly interspaced short palindromic repeats (CRISPR/CRISPR-associated protein 9 [Cas9]) gene editing libraries that use single-guide RNAs to knock-out target SLC genes to interrogate biological responses.^39^ The use of this library allows investigation into the role an unknown SLC transporter may play in the uptake of a particular drug of interest in a translationally relevant cell line in an unbiased manner that is compatible with large-scale testing. This approach provides a high-throughput readout based on drug-induced cytotoxicity, uptake (eg, radioactivity or fluorescence), and/or interaction with an intracellular biosensor. CRISPR/Cas9 knockout or siRNA knockdown techniques can narrow down a broad list of transporters to the most promising targets for further functional verification and validation.^40^^,^^41^ Alternative methods to direct uptake assays include competitive counterflow assays, which use a radiolabeled or fluorescent, prototypical substrate for a transporter of interest.^42^, ^43^, ^44^ In the competitive counterflow assay, the substrate (probe) is incubated until steady state, and then, the test drugs are evaluated for their substrate interaction with the transporter based on their ability induce probe efflux from the cells. These methods avoid the need for expensive and sometimes inaccessible radiolabeled drugs of interest, offering a more convenient and high-throughput method of investigating the substrate repertoire of a given transporter. Bridging the gap between in vitro and proper ex vivo experimentation, elegant systems such as the “organ-on-a-chip” and human-induced pluripotent stem cell–derived cells yield models that may retain biological complexity and toxicological relevance better than traditionally engineered, overexpressed cell lines.^45^, ^46^, ^47^, ^48^ These models have the potential to facilitate our mechanistic understanding of drug transport and toxicity in clinically relevant and patient-specific contexts.^49^, ^50^, ^51^

In addition to cell-based investigation, the availability of transporter-deficient in vivo models for most translationally relevant SLCs has greatly advanced investigations into their connection with drug-induced organ injury. Beyond the commonly used animal models genetically deficient (globally) for a given transporter,^52^ conditional knock-in or knock-out animals are now widely available, which allows for the deletion or insertion of genes with tissue or cell-type specificity.^53^ Models like these can help disentangle the impact of an SLC in 1 tissue from its body-wide contribution to the disposition of a drug. Moreover, the existence of humanized rodent models for various xenobiotic transporters allows for the in vivo exploration of the activity of the human protein, rather than its rodent ortholog.^54^^,^^55^ Such examples include the generation of a cluster knock-in of human OATP genes into a mouse with a global deficiency of the murine OATPs, which may provide more translationally relevant predictive models for the interaction of xenobiotics with human transporters.^56^ This is especially important in understanding the contribution of SLCs to the pharmacokinetics and tissue distribution of xenobiotics, given that the transport kinetics of various substrates and the sensitivity to inhibition by xenobiotics are species-dependent.^57^

In addition to rodents, zebrafish have emerged as a suitable, alternative model organism commonly used in drug toxicity research focused on evaluating the functional consequences of genetic or pharmacological manipulation of transporters.^58^, ^59^, ^60^ Zebrafish offer several key advantages over rodents or even larger species, including the fact that they are easily amenable to genetic manipulation and that their physical transparency allows for optical in vivo fluorescent imaging. Moreover, zebrafish are relatively small and highly reproductive, thereby enabling high-throughput genetic and functional screens with translational utility for modeling cancer and other diseases.^59^

The large-scale, unbiased testing or detection of chemical species in the form of high-throughput screening is a cornerstone of drug discovery and continues to expand in applicability and accessibility.^61^ Biologically relevant assays that can be performed quickly and inexpensively ensure that contributions can be made to the understanding of the interactome of a given enzyme, transporter, or signaling pathway. This screening method allows for the use of large libraries of compounds to ensure a lack of bias in the agents chosen for the assay. Similar advancements have been made in metabolomics, transcriptomics, and proteomics.^62^ Metabolomics refers to the sampling of biological material between experimental groups and subsequent analyses to identify uniquely altered endogenous or even exogenous metabolites in a “targeted” or “untargeted” fashion.^63^ Metabolomics studies are imperative in the discovery and investigation of new biomarkers for disease states and tumor targets.^64^ As such, metabolomics has provided a systemic measure of an inhibitor drug on an endogenous substrate concentration as a readout for pharmacokinetic drug-drug interaction liabilities for many clinically relevant transporters, including OATs, OCTs, and OATPs. In addition, metabolomics can also provide a readout on metabolites that are essential for normal tissue homeostasis and off-target effects of transporter inhibition. This approach mirrors targeted and untargeted proteomics in its capacity for the identification of tissue expression of transporters.^65^ The ability to reliably detect and quantify protein expression and abundance of an SLC, in addition to the measurement of transcripts, with mass spectrometry–based proteomics approaches now allows for quantitative readouts not previously possible with western blots.^66^ In addition, expression analyses have become increasingly sophisticated and represent a powerful analytical tool. Investigators can rapidly evaluate SLC expression and pathway signatures in tissues of interest with the use of single-cell RNA-sequencing as well as spatial transcriptomic or proteomic approaches.^67^, ^68^, ^69^, ^70^ These elegant analyses offer the possibility of cell-specific and spatially oriented tissue investigation, and with advances in artificial intelligence and machine learning, the capabilities of these approaches can improve the quantitative and qualitative understanding of SLC transporter expression and systems toxicology. These practices are revolutionizing not only the clinical investigation of biomarkers but also the preclinical investigation of model organisms to verify translational relevance and identify any potential discrepancies or confounding factors that may compromise therapeutic potential in humans.

## II Solute carrier transporters and drug-induced toxicity

### A Platinum drugs

Platinum-based drugs, which include cisplatin, carboplatin, and oxaliplatin, are widely used to treat various solid tumors. Cisplatin was the first of the platinum compounds to be used in the clinic, after a serendipitous discovery in inhibiting the cell division of *Escherichia coli,* and was then shown to affect rapidly dividing cancer cells.^71^^,^^72^ Although they share a similar mechanism of action, the clinical indications and safety profiles of cisplatin, carboplatin, and oxaliplatin differ vastly. For cisplatin, acute kidney injury (nephrotoxicity), hearing loss (ototoxicity), and nausea/vomiting are the most frequently observed severe side effects, whereas carboplatin and oxaliplatin are associated with a high incidence of reduced platelet counts (thrombocytopenia) and injury to peripheral nerves (neurotoxicity), respectively.^73^ In addition to differences in their safety profile, the platinum-based chemotherapeutics do not share many overlapping indications, thereby limiting alternative therapeutic options in the case of treatment discontinuation or disease progression. Besides cisplatin, carboplatin, and oxaliplatin, there are novel platinum derivatives and complexes under investigation that aim to reduce unwanted toxicities and avoid acquired resistance mechanisms occurring with the approved platinum drugs.^74^ The recognition that certain SLC transporters play a pivotal role in the cellular accumulation of platinum drugs occurred after their initial use in the clinic.^34^^,^^75^^,^^76^

#### 1 Cisplatin-induced nephrotoxicity

Cisplatin is the most broadly utilized platinum drug in cancer contexts and is considered to be the most nephrotoxic of the group,^77^ although injury to the kidney can still occur with the use of oxaliplatin and carboplatin.^78^ Cisplatin-associated nephrotoxicity causes an immense burden on the healthcare system and can dramatically impact a patient’s quality of life, as the preservation of kidney function during treatment with cisplatin is often justification for dose reduction or treatment discontinuation. A summary of SLC transporters capable of transporting platinum drugs is included in Table 1.^79^, ^80^, ^81^, ^82^, ^83^, ^84^, ^85^, ^86^, ^87^, ^88^, ^89^, ^90^, ^91^, ^92^, ^93^, ^94^, ^95^, ^96^, ^97^, ^98^, ^99^, ^100^, ^101^, ^102^, ^103^, ^104^, ^105^, ^106^, ^107^, ^108^, ^109^, ^110^, ^111^, ^112^, ^113^, ^114^, ^115^, ^116^, ^117^, ^118^, ^119^, ^120^, ^121^ Of these transporters, the cationic transporters OCT2 (SLC22A2), MATE1 (SLC47A1), and MATE2K (SLC47A2) are highly expressed in renal proximal tubular cells and collectively, as a functional unit, mediate the uptake of cisplatin from the blood into tubular cells (OCT2) and its efflux into the urine (MATE1 and MATE2K).^28^^,^^79^^,^^80^^,^^122^, ^123^, ^124^ Cisplatin-induced nephrotoxicity can largely be attributed to the ability of cisplatin to accumulate in proximal tubular epithelial cells in an OCT2-dependent manner and can be exacerbated if MATE1- and MATE2K-mediated efflux is impaired. In support of this supposition, the substrate affinity of each platinum drug can partially explain the differences in the prevalence and severity of nephrotoxicity. In particular, carboplatin is not considered a transported substrate of OCT2 and predominantly undergoes glomerular filtration, whereas oxaliplatin is an OCT2 substrate but is more efficiently removed from tubular cells by MATE1 and MATE2K than cisplatin.^81^

**Table 1:** Toxicities and SLC interactions of cytotoxic chemotherapy

Drug	Toxicity^a^	Transporters^b^	References^c^
Platinum Drugs			
Cisplatin	Gastrointestinal Myelosuppression Nephrotoxicity Neurotoxicity Ototoxicity	CTR1, MATE1, MATE2K, OAT1, OAT3, OATP1B1, OATP1B3, OCT1, OCT2, OCT3, OCTN1, and OCTN2	^76^^,^^79^, ^80^, ^81^, ^82^, ^83^
Oxaliplatin	Cardiotoxicity Gastrointestinal Hepatotoxicity Myelosuppression Neurotoxicity	CTR1, MATE1, MATE2K, OATP1B3, OCT2, OCT3, OCTN1, and OCTN2	^76^^,^^81^, ^82^, ^83^
Carboplatin	Gastrointestinal Myelosuppression Nephrotoxicity Neurotoxicity Ototoxicity	CTR1 and OATP1B3	^82^^,^^83^

Nucleoside Analogs			
Azacitidine	Gastrointestinal Myelosuppression	CNT1, CNT2, CNT3, ENT1, ENT2, ENT3, and ENT4	84, 85, 86
Cladribine	Myelosuppression	CNT2, CNT3, ENT1, ENT2, and ENT4	^87^^,^^88^
Clofarabine	Myelosuppression	CNT2, ENT1, ENT2, and OCTN1	^87^^,^^89^
Cytarabine	Gastrointestinal Myelosuppression	ENT1, ENT2, CNT1, OCTN1, and MATE1	^87^^,^^89^^,^^90^
Decitabine	Gastrointestinal Myelosuppression	ENT1 and ENT2	^85^
Fludarabine	Central nervous system Myelosuppression	CNT3, ENT1, ENT2, and OCTN1	^87^^,^^89^^,^^91^
Gemcitabine	Gastrointestinal Myelosuppression	CNT1, CNT3, ENT1, ENT2, ENT3, ENT4, OCTN1, and MATE1	^87^^,^^89^^,^^92^
Nelarabine	Central nervous system Gastrointestinal Myelosuppression	ENT1 and ENT2	^93^^,^^94^
5-fluorouracil	Gastrointestinal Myelosuppression Neurotoxicity	ENT1, ENT2, CNT1, CNT2, MATE1, and OAT2	^88^^,^^95^, ^96^, ^97^, ^98^
6-mercaptopurine	Gastrointestinal Myelosuppression	ENBT1, ENT1, ENT2, CNT2, and CNT3	99, 100, 101, 102

Anthracyclines			
Daunorubicin	Cardiotoxicity Gastrointestinal Myelosuppression	OATP1B1, OCT1, and OCTN1	103, 104, 105
Doxorubicin	Cardiotoxicity Gastrointestinal Hepatotoxicity Oral mucositis	OATP1A2, OCT1, OCT2, OCT3, OCT6, OCTN1, and OCTN2	106, 107, 108, 109
Epirubicin	Cardiotoxicity Gastrointestinal Myelosuppression Oral mucositis	OCT1	^110^
Idarubicin	Cardiotoxicity Myelosuppression	--	^87^
Mitoxantrone	Cardiotoxicity Myelosuppression	OCTN1	^111^
Valrubicin	Gastrointestinal	--	^112^

Vinca Alkaloids			
Vinblastine	Gastrointestinal Myelosuppression	--	^113^
Vincristine	Gastrointestinal Myelosuppression Peripheral neuropathy	OATP1B1, OATP1B3, OATP2B1, OATP1C1, OCT3, and MATE1	113, 114, 115
Vindesine	Myelosuppression Peripheral neuropathy	--	^113^
Vinflunine	Gastrointestinal Myelosuppression Neurotoxicity	--	^116^
Vinorelbine	Gastrointestinal Myelosuppression	--	^117^

Taxanes			
Cabazitaxel	Gastrointestinal Myelosuppression Peripheral neuropathy	OATP1B3	^118^
Docetaxel	Gastrointestinal Myelosuppression Peripheral neuropathy	OATP1A2, OATP1B1, OATP1B3, and OATP1C1	^119^
Paclitaxel	Cognitive impairment Gastrointestinal Myelosuppression Peripheral neuropathy	OATP1A2, OATP1B1, and OATP1B3	^98^^,^^120^^,^^121^

a Listed toxicities were identified based on the FDA insert information, alphabetically.

b Interaction of cytotoxic chemotherapy with SLCs as substrates.

c References for toxicity or interaction.

In rodents that are genetically deficient for Oct1 and Oct2, which are jointly orthologous to human OCT2, or in animals receiving concurrent treatment with OCT2 inhibitors, the kidneys are partially protected against cisplatin-induced nephrotoxicity. In contrast, knockout or pharmacological inhibition of Mate1, the single murine ortholog of MATE1 and MATE2K in humans, can exacerbate the injury.^80^^,^^81^ In addition to cation transporters, the anion transporters OAT1 (SLC22A6) and OAT3 (SLC22A8) are also partially responsible for cisplatin-induced nephrotoxicity. In rodents, Oat1 and Oat3 are capable of transporting the toxic mercapturic acid metabolite of cisplatin, a negatively charged N-acetylcysteine S-conjugate, which induces renal tubular injury in a p53-dependent but OCT2-independent manner.^125^^,^^126^ Recent studies have suggested that the renal expression of Mate1 and Oct2 is under the control of the peroxisome proliferator-activated receptor alpha (PPAR-*α*) transcription factor, which could thus potentially serve as a therapeutic target to mitigate cisplatin-induced nephrotoxicity. In particular, genetic deficiency of PPAR-*α* is associated with increased Mate1 expression and decreased Oct2 protein abundance in mouse proximal tubular cells, implying that pharmacological inhibition of PPAR-*α* is expected to reduce the accumulation of cisplatin in the kidney.^127^

Beyond OCT2 and MATE1, the copper transporter (CTR)1 (SLC31A1) has been implicated in the accumulation of cisplatin in the kidney.^122^ However, the protective effects of Ctr1 knockouts are less pronounced than Oct1/Oct2 knockouts, suggesting that CTR1 might only be partially contributing to cisplatin transport in the kidney. The clinical translation of targeting this pathway for cisplatin-induced nephrotoxicity remains uncertain, as CTR1 is also detectable in multiple cisplatin-sensitive tumors, including ovarian cancer,^128^ and intentional modulation of CTR1 may thus affect the therapeutic efficacy of cisplatin.^82^ It should also be pointed out that since multiple uptake transporters contribute to the initiating pathophysiology of cisplatin-induced nephrotoxicity, antagonism of 1 single pathway may not offer complete protection from this injury. Thus, the identification of small-molecule inhibitors that can simultaneously target these pathways without antagonizing the therapeutic activity of cisplatin is required before such a strategy can be considered an effective adjunct therapy for the prevention of this injury.^125^

#### 2 Cisplatin-induced ototoxicity

Cisplatin-induced hearing loss is another debilitating and well-documented clinical adverse event, especially in patients with testicular cancer.^129^ Because cochlear sensory hair cells within the inner ear do not regenerate, this damage is often considered to be irreversible and progressive with cumulative doses. This ototoxicity affects nearly half of patients receiving cisplatin and is a burden on the patients’ quality of life, especially considering long-lasting effects in children and young adults.^130^ Current clinical options for the mitigation of this phenomenon are insufficient for the challenge it poses; reductions in dose or cessation of cisplatin treatment, especially in patients deemed at higher risk, are currently the best ways to avoid cisplatin ototoxicity.^131^ Interestingly, transporters involved in cisplatin-induced nephrotoxicity, including OCT2, CTR1, and MATE1, are also expressed on cochlear sensory hair cells and have been implicated in the initiation of ototoxicity in animal models.^132^, ^133^, ^134^ A role of OCT2 in cisplatin-induced ototoxicity is consistent with pharmacogenetic studies, implying that a common single-nucleotide polymorphism (SNP) in *SLC22A2* (rs316019) is significantly associated with protection against this injury in a retrospective and exploratory analysis in both pediatric and adult patients. This *SLC22A2* variant, which corresponds to the nucleotide substitution at position 808G>T, was associated with reduced transport of prototypical substrates, suggesting that the reduced transport affinity for OCT2 substrates can reduce accumulation and protect cochlear sensory hair cells against the deleterious effects of cisplatin.^135^ It is noteworthy that in an attempted replication study, this variant was not significantly associated with the severity of ototoxicity, thus implying that large-scale, prospective studies are warranted to confirm a connection of variant OCT2 genotypes with this side effect.^136^ Similarly, patients with lung cancer carrying a SNP in *SLC31A1* (rs10981694) were at increased risk of severe ototoxicity.^137^ However, in the above retrospective and exploratory analysis of pediatric and adult cancer patients receiving cisplatin, which identified *SLC22A2* rs316019 as having protective effects, *SLC31A1* rs10981694 was not associated with increased severity of cisplatin-induced ototoxicity.^138^

In addition to OCT2 and CTR1, the monocarboxylate transporter 6 (MCT6; SLC16A5) has been implicated as a contributor to cisplatin ototoxicity. Although the role of MCT6 in mediating cisplatin transport is unclear, it was identified as a potential risk factor for cisplatin-induced hearing loss in a genome-wide association study in patients with testicular cancer through a mechanism that may involve MCT6-dependent accumulation of the toxic cisplatin metabolite N-acetylcysteine S-conjugate in cochlear sensory hair cells.^139^ However, follow-up studies have not confirmed MCT6 as a risk factor contributing to cisplatin-induced hearing loss.^138^^,^^140^^,^^141^ Interestingly, genetic deficiency of the related transporters Mct8 (Slc16a2) and Mct10 (Slc16a10) in mice was associated with progressive degeneration of cochlear hair cells and loss of endocochlear potential.^142^ This suggests that, at least in part, this class of transporters may play a role in regulating tissue homeostasis and injury to cisplatin within the ear. Collectively, although replication failures can occur due to the heterogeneity in population composition, sample size, and complexities in biological pathways, the use of these variants (SLC22A2, SLC31A1, and SLC15A5) as pharmacogenetic biomarkers for cisplatin-induced ototoxicity warrants further attention in larger patient cohorts.

#### 3 Oxaliplatin-induced neurotoxicity

In patients receiving oxaliplatin, up to 90% of patients experience oxaliplatin-induced peripheral neurotoxicity,^143^ an incidence that is much higher than that observed with cisplatin.^144^^,^^145^ Damage to the peripheral nervous system by these agents can be acute, occurring immediately after infusion, or chronic, lasting for years after discontinuation or completion of therapy.^146^ It has been suggested that oxaliplatin may be transported in the rat dorsal root ganglion (DRG), the primary site of oxaliplatin accumulation within the nervous system, by the ergothioneine transporter novel organic cation transporter (OCTN)1 (SLC22A4), and, to a lesser extent, by the carnitine transporter OCTN2 (SLC22A5).^147^ However, OCTN1-mediated transport of oxaliplatin could not be confirmed in follow-up investigations,^148^ and Octn1 deficiency in mice did not afford neuroprotection.^149^ It is conceivable that the neuroprotective effects associated with physiological concentrations of ergothioneine in chronic oxaliplatin treatment regimens are partially due to effects on transporters other than OCTN1.^150^ Support for this comes from a prior study demonstrating that ergothioneine can significantly reduce the cellular uptake of oxaliplatin by more than 50% in DRG neurons from Octn1-deficient mice.^147^ Interestingly, impairment of Mate1 function by siRNA in rats exacerbated peripheral neurotoxicity,^151^ although a direct role for this transporter in the mammalian DRG in response to oxaliplatin remains unclear.^149^ Multiple preclinical studies have suggested that deficiency of Oct2, a transporter expressed in satellite glial cells within DRG, in mice and rats provides protection against oxaliplatin and cisplatin neurotoxicity due to an accumulation defect and that administration of OCT2 inhibitors can phenocopy such phenomena.^149^^,^^152^, ^153^, ^154^ The translational feasibility of OCT2 inhibition by dasatinib, a potent noncompetitive inhibitor of OCT2, for the prevention of oxaliplatin-induced neurotoxicity in patients with colorectal cancer is currently being explored in a phase 1b study (NCT04164069).

A drug that has received National Comprehensive Cancer Network recommendation for chemotherapy-induced peripheral neurotoxicity is the serotonin-norepinephrine reuptake inhibitor duloxetine, although its use in connection with oxaliplatin has been largely explored in the context of symptom management rather than as a preventative strategy. It is worth pointing out in this context that duloxetine was previously identified from a high-throughput drug library screen as an inhibitor of OCT2.^153^ In a follow-up study, duloxetine was confirmed to potently inhibit OCT2, and pretreatment with duloxetine in mice protected against oxaliplatin neurotoxicity without affecting its antitumor properties.^155^ This observation supports the idea that the efficacy of duloxetine in clinical pain management for patients receiving oxaliplatin can partially be attributed to OCT2 inhibition.^156^ The presence of CTR1 in neurons has also been suggested to enhance oxaliplatin damage in rat models.^157^ Although this corroborates in vitro evidence that CTR1 mediates the uptake of platinum drugs, including oxaliplatin,^82^ more recent studies employing CRISPR-mediated knockout of CTR1 have demonstrated that oxaliplatin accumulation in rat DRG occurs independently of CTR1.^158^ Although carboplatin rarely causes peripheral neurotoxicity in humans, it can cause neurotoxic phenotypes in rats, especially in the context of its use with other neurotoxic agents like paclitaxel.^159^ The transport mechanisms involved in this process, however, and the contribution of CTR1 to this process remain unclear. Because the organic anion transporting polypeptides OATP1B1 (SLCO1B1) and OATP1B3 (SLCO1B3) have been implicated in mediating the uptake of cisplatin, carboplatin, and oxaliplatin in some cells,^83^ it is possible these mechanisms contribute to the accumulation of carboplatin in DRG.

### B Nucleoside analogs

Nucleoside analogs are a class of commonly used anticancer agents that include azacitidine, cytarabine, decitabine, gemcitabine, 6-mercaptopurine, and 5-fluorouracil (5-FU). The mechanisms of action of drugs in this class are diverse and can induce cytotoxicity ranging from interfering with DNA synthesis (cytarabine) to the inhibition of enzymes such as DNA methyltransferase (azacitidine and decitabine) or thymidylate synthase (5-FU).^84^^,^^160^^,^^161^ Although this is an efficacious strategy against highly proliferative cells like cancer cells, normal healthy cells that are highly proliferative are often equally vulnerable to this mechanism,^87^ including cells within the bone marrow. Nucleoside analogs are frequently used in the context of hematological malignancies, including acute and chronic leukemias, as well as some solid tumors in the case of gemcitabine and 5-FU.^161^^,^^162^ In terms of transport, these drugs are known to interact with concentrative nucleoside (CNT) and equilibrative nucleoside (ENT) transporters, which are encoded by the *SLC28* and *SLC29* subfamilies, respectively.^85^^,^^87^^,^^95^^,^^163^^,^^164^ The interactions of nucleoside analogs with SLC transporters are summarized in Table 1. CNTs are considered high-affinity, sodium-dependent, and inwardly directed, whereas the ENTs are considered low-affinity diffusion facilitators, and are bidirectional.^165^, ^166^, ^167^ In addition to traditionally recognized nucleoside transporters, OCTN1 was found to be a high-affinity transporter for cytarabine, gemcitabine, clofarabine, and fludarabine.^89^ Furthermore, cells overexpressing OCTN1 displayed increased sensitivity to these drugs, and the expression of OCTN1 was positively correlated with response in patients with acute myeloid leukemia receiving cytarabine-based chemotherapy.^168^ The interaction of cytarabine with OCTN1 is consistent with reports indicating that several other cation transporters, including MATE1, can transport several of these agents.^90^ Some investigators, however, have forcefully argued that OCTN1 is a high-affinity transporter of ergothioneine that does not meaningfully contribute to the cellular uptake of any xenobiotics, including nucleoside analogs.^169^^,^^170^

Myelosuppression is one of the most prevalent and severe toxic effects of nucleoside analog treatment,^84^^,^^99^^,^^171^ although some neurological toxicities have been reported as well.^93^^,^^96^^,^^172^^,^^173^ Myelosuppression can result in reduced levels of red blood cells (anemia), thrombocytopenia, loss of neutrophils (neutropenia), and other hematological disorders that can affect a patient’s well-being.^174^^,^^175^ The clinical sequelae of these blood cell abnormalities include severe hemorrhages and opportunistic infections, associated with a weakened immune system, which can lead to life-threatening complications such as sepsis and death. The myelosuppressive effects of 6-mercaptopurine, used for the treatment of acute lymphoblastic leukemia in pediatric patients, were attributed to altered metabolism and subsequent increases in systemic exposure.^176^ Such findings give credence to the hypothesis that, in addition to directly contributing to uptake in the target malignant cells, SLCs affecting xenobiotic elimination pathways can affect measures of systemic drug exposure and induce toxic effects. It is also worth noting that agents like decitabine can alter the methylation status of genes, and therefore, the function of certain transporters of relevance to chemotherapeutic drugs.^177^, ^178^, ^179^

#### 1 Nucleoside analog–induced myelosuppression

The bone marrow is the site of hematopoiesis, which serves to replenish all types of circulating blood cells.^180^ The population of bone marrow cells includes neutrophils, macrophages, monocytes, and natural killer cells, as well as other precursor cells.^181^^,^^182^ The injury to the bone marrow induced by these drugs has been known since their initial use^183^ and is especially prominent in combinatorial regimens with azacitidine and cytarabine or decitabine and the BCL2 inhibitor venetoclax.^86^^,^^184^ The clinical sequelae of myelosuppression commonly require the usage of antifungals and antibiotics, many of which having been implicated themselves as perpetrators of drug-drug interactions involving both metabolizing enzymes and transporters.^185^, ^186^, ^187^

ENTs and CNTs that mediate the uptake of nucleosides are highly expressed in bone marrow and the hematopoietic stem cells that differentiate into cell lineages relevant to injury induced by nucleoside analogs.^188^^,^^189^ Evidence for differential myelosuppressive effects with the use of 6-mercaptopurine was identified from SNPs in genes encoding for CNT and ENT transporters. Specifically, wild-type *SLC29A1* rs747199 (CC) was associated with a lower risk of agranulocytosis and leukopenia compared with (CG) and (GG) genotypes, *SLC28A2* rs2431775 was associated with worsened neutropenia, and S*LC28A3* rs10868138 was associated with worsened leukopenia.^190^ In further support, the microenvironment of the bone marrow niche, including stromal cells, in murine models was shown to downregulate the function of Ent1 (Slc29a1). This downregulation protected leukemia cells from cytarabine, implying a contribution of ENT1 as a mediator of drug efficacy.^191^ Beyond this, the absence of ENT1 has been linked to poor survival in patients with pancreatic cancer receiving gemcitabine,^192^ and loss of ENT1 during epithelial-mesenchymal transition is correlated with gemcitabine resistance in pancreatic cancer cells.^193^

Further demonstrations of SLC interactions with nucleoside analogs exist in the published literature dealing with drug resistance. For example, resistance to the immunosuppressant azathioprine, a prodrug of 6-mercaptopurine, has been associated with expression changes in ENT1, ENT2 (SLC29A2), and CNT3 (SLC28A3).^99^^,^^100^ In addition, 6-mercaptopurine has been identified as a substrate of equilibrative nucleobase transporter (ENBT)1, encoded by SLC43A3, and ENBT1 function was shown to modulate 6-mercaptopurine transport and cytotoxicity in models of leukemia.^101^^,^^102^^,^^194^ Similarly, in leukemic models of cytarabine resistance, downregulation of ENT1 was associated with reduced cellular sensitivity.^195^ Resistance in leukemia cells to 6-thioguanine has been attributed to the downregulation of ENT3 (SLC29A3) and CNT3, and the introduction of CNT2 into drug-resistant leukemia cells restored sensitivity to 5-FU.^88^^,^^196^ Additionally, a significant correlation has been documented between low expression of CNT1 (SLC28A1) and poor response to cladribine (2-chloro-2’-deoxyadenosine).^197^ The authors speculated that the expression of nucleoside transporters can be used prospectively to determine the therapeutic activity of treatment with nucleoside analogs. A proof-of-concept study supports this in that the renal elimination of gemcitabine was increased and associated with poor antitumor responses in Cnt1-deficient mice.^198^ These reports collectively support the notion that SLC expression in tumors could be used as a biomarker for efficacy and that SLC expression in sites of injury, such as the bone marrow, could be used as a predictor for toxicity.

### C Anthracyclines

Anthracyclines are a class of widely used chemotherapeutics that were discovered in the mid-20th century. They are utilized for the treatment of a variety of cancers, including hematological malignancies, breast cancer, and soft tissue sarcomas.^199^ The anthracycline class includes drugs such as daunorubicin, doxorubicin, epirubicin, idarubicin, and valrubicin, whereas mitoxantrone is an anthraquinone.^200^ The mechanism of action of anthracyclines is considered to be multifactorial and includes the generation of reactive oxidative species and free radicals, inhibition of DNA synthesis, inhibition of topoisomerase II, and ferroptosis activation in target cells. These mechanisms can contribute to antitumor activity as well as damage in normal tissues.^200^, ^201^, ^202^, ^203^ Indeed, these pathways of cell damage can help explain the long list of severe adverse effects recorded with the use of anthracyclines, which include gastrointestinal (GI) irritation and vomiting, myelosuppression, inflammation of the mouth and digestive tract (mucositis), hair loss (alopecia), hepatotoxicity, and cardiotoxicity.^204^, ^205^, ^206^, ^207^, ^208^, ^209^, ^210^, ^211^ These adverse effects are a burden on the long-term health of patients, especially pediatric patients who might suffer from late-onset cardiotoxicity several years after the cessation of treatment.

#### 1 Doxorubicin-induced cardiotoxicity

Cardiotoxicity is considered the most noteworthy side effect from anthracycline treatment,^212^ especially with doxorubicin. Upon development of congestive heart failure, it is estimated that mortality can be as high as ∼50%.^213^ Anthracycline-induced cardiotoxicity is distinctly exposure-dependent, and it is estimated that about 6% of patients suffer from “overt” cardiotoxicity; however, nearly a quarter of patients treated with anthracyclines may suffer some form of clinical or subclinical cardiotoxicity.^214^^,^^215^ The cumulative exposure limit for doxorubicin patients is between 450 and 550 mg/m^2^ body surface area. The mechanism of cardiotoxicity induced by doxorubicin and other anthracyclines is not completely understood and is still undergoing extensive investigation but is hypothesized to utilize several of the same pathways as described above that are involved in the mechanism of action against tumor cells.^216^ However, there is recognition that most of the pathways associated with injury are dependent on the ability of doxorubicin to access intracellular compartments. Current tools to prevent or treat doxorubicin-induced cardiotoxicity (DIC) are generally lacking in efficacy or have failed to demonstrate clinical effectiveness and remain inadequate to address the clinical challenges that this side effect poses.^217^, ^218^, ^219^ Dexrazoxane is currently the only FDA-approved agent to prevent DIC, although investigations into its use as a cardioprotectant have offered mixed results.^220^ Concerns regarding the use of dexrazoxane include the notion that it can cause myelosuppression, that it may reduce the antitumor efficacy of doxorubicin, and that it may cause secondary malignancies, especially in pediatric populations.^221^^,^^222^ Aside from dexrazoxane, the use of angiotensin-converting enzyme inhibitors and *β*-blockers has shown some utility in protecting against DIC, although more thorough investigation into this strategy is warranted.^223^

Doxorubicin is reported to be a transported substrate for several SLCs, as summarized in Table 1. Using a variety of in vitro models, including engineered overexpressing cell lines, cancer cell lines, and stem cell–derived cardiomyocytes, SLCs that have been proposed to transport doxorubicin include OCT1 (SLC22A1), OCT2, OCT3 (SLC22A3), OCT6 (SLC22A16), OCTN1, OCTN2, CNT3, OATP1A2 (SLCO1A2), OATP1B1 (SLCO1B1), and OATP1B3 (SLCO1B3).^106^, ^107^, ^108^, ^109^^,^^224^

In cardiomyocytes originating from pluripotent stem cells (hiPSC-CMs) isolated from pediatric patients treated with doxorubicin, OCT3 was identified as an upregulated transporter in those patients who suffered from DIC.^109^ These studies revealed that the deficiency of Oct3 by genetic knockout yields protection from this injury in mice without concurrent changes in the plasma pharmacokinetic profile of doxorubicin. This protection could be phenocopied in wild-type mice by pretreatment with nilotinib, a pharmacological inhibitor of Oct3. The observation that nilotinib can prevent DIC in rodents was first reported in a rat model of DIC through an unknown mechanism,^225^ a finding that could now be attributed to inhibition of Oct3. Interestingly, a separate study showed that nilotinib, when used at high doses, can actually exacerbate DIC.^226^ The differential effect of nilotinib to protect or exacerbate DIC is likely due to the overlapping cardiotoxic potential of doxorubicin and nilotinib, and/or the ability of nilotinib to inhibit ABCB1 (P-glycoprotein)-mediated efflux at high doses.^16^ In addition, OCT6 was also identified as an upregulated transporter in the same hiPSC-CMs from patients with DIC,^109^ corroborating the earliest report of doxorubicin transport by OCT6 using *Xenopus oocytes* overexpression and a leukemia model with constitutive expression of OCT6.^224^ Therefore, future mechanistic studies aimed at evaluating the role of OCT6 in the development of DIC are warranted.

Furthermore, it has been reported that doxorubicin is a substrate of Octn1 and Octn2, and inhibition by high-dose metformin is associated with cardioprotection in a mouse model of DIC.^227^ A separate study also demonstrated that ergothioneine, a substrate of OCTN1, is also capable of protecting against DIC.^228^ OCTN1 was also an upregulated transporter in hiPSC-CMs with DIC, in the same samples as OCT3 and OCT6, and genetic knockout of OCTN1 in patient-derived cardiomyocytes was associated with reduced doxorubicin uptake and an increase in cell viability.^107^ The utility of metformin as a preventative strategy for DIC is in accordance with other reports,^229^^,^^230^ although this has not been corroborated in other studies. For example, a rat model of DIC did not identify cardioprotective effects by metformin, despite promising in vitro findings,^231^ and metformin was unable to reduce the onset and severity of DIC in breast cancer patients receiving metformin prior to doxorubicin administration (NCT02472353).^232^

In addition to organic cation transporters, a SNP in the CNT3 gene *SLC28A3* (rs7853758) has been identified to be significantly associated with an increase in the odds of DIC.^233^^,^^234^ The relevance of this SNP was subsequently verified by observations that the expression of CNT3 was upregulated in hiPSC-CM from patients who suffered from DIC.^201^ Further verification included findings that genetic perturbations showed protective effects from cellular hallmarks of DIC in vitro and that the use of desipramine, identified from a high-throughput library screen aimed at identifying a cardioprotective agent, conferred protection in vivo.^107^ Because desipramine is also an inhibitor of cationic transporters,^235^ follow-up studies with Cnt3-deficient in vivo models and CNT3 uptake studies with desipramine are warranted to confirm a direct role of this transporter in DIC. It was recently reported that the use of empagliflozin, an inhibitor of the sodium-glucose cotransporter 2 (SGLT2 and SLC5A2), can attenuate DIC in hiPSC-CMs and rodent models and protect against heart failure induced by doxorubicin in patients.^236^^,^^237^ However, these studies do not provide a definitive demonstration that the observed protection is dependent on cardiac SGLT2 or transport of doxorubicin by SGLT2.^238^ Further studies are also warranted to determine if empagliflozin can inhibit doxorubicin transporters in the heart.

The use of SLC inhibitors may hold promise for use with doxorubicin and other anthracyclines to ameliorate cardiotoxicity by preventing excessive accumulation in cardiomyocytes, although such a strategy has not yet been employed successfully in patients. The clinical implementation of a transport inhibitor-based cardioprotective strategy requires the use of a potent, selective inhibitor of doxorubicin accumulation for at least the duration of the anthracycline infusion. The proper dosage, timing, pharmacokinetic profile, and lack of deleterious effects in other tissues would need to be optimized and supported by strong evidence. It is important to mention that liposome-encapsulated doxorubicin is now available for clinical use, and the rate of cardiotoxicity in patients receiving this product is lower compared with that observed with unencapsulated doxorubicin.^239^^,^^240^ However, liposomal encapsulation of drugs, although increasingly common, does not erase the toxicity profile of the delivered cancer drug and might possibly yield its own toxicity concerns.^241^^,^^242^

#### 2 Anthracycline-induced hepatotoxicity

In addition to the notable cardiotoxicity, doxorubicin is known to cause hepatotoxicity in some patients, and dose modifications are suggested in the prescribing information for patients with hepatic impairment.^243^^,^^244^ There is evidence to suggest that patients with hepatic impairment display altered pharmacokinetics of doxorubicin, although this does not seem to cause an increased risk of adverse events as treatment progresses.^245^ Doxorubicin has also been identified as a substrate for OATP1A2 and OATP1B transporters even though it is a hydrophobic weak base. As such, genetic deficiency of all Oatp1a and Oatp1b isoforms in mice was associated with increased plasma concentrations and reduced exposure of the liver compared with wild-type mice.^246^^,^^247^ In the same model, the transgenic liver-specific expression of the human isoforms OATP1A2, OATP1B1, and OATP1B3 was able to partially rescue doxorubicin plasma concentrations to levels observed in wild-type mice.^246^ These observations are in line with the finding that the related anthracycline daunorubicin is a transported substrate of OATP1B1 and its murine ortholog Oatp1b2. Similarly, measures of systemic exposure to daunorubicin were increased in Oatp1b2-null animals.^103^ Direct hepatotoxicity induced by doxorubicin in mice has also been linked with transport into hepatocytes by Octn1 and Octn2,^108^ and inhibition of these 2 transporters by metformin was observed to ameliorate hepatotoxicity in this preclinical model. Daunorubicin and doxorubicin are also known to be transported efficiently by the hepatocellular transporter OCT1,^106^ and increased expression of OCT1 in ovarian cancer was associated with improved survival in patients.^104^ Given these observations, it is conceivable that transport by OCT1 may also mediate the hepatic uptake of anthracyclines and contribute to toxicity, although additional investigations are required to substantiate this hypothesis.

#### 3 Anthracycline-induced gastrointestinal toxicity and mucositis

Oral mucositis is another common adverse event related to anthracycline treatment and can have a profound, deleterious impact on patients’ quality of life.^248^^,^^249^ Xenobiotic transporters are known to be expressed in the GI tract and salivary glands.^250^ However, a definitive connection between SLC transport of anthracyclines and oral mucositis is unknown.^249^^,^^251^^,^^252^

Apart from oral mucositis, anthracycline treatment can cause generalized GI toxicity such as nausea and vomiting. These irritations are assumed to be unavoidable with the administration of chemotherapy, but specific mechanisms of intestinal damage induced by doxorubicin have been investigated using intestinal organoids.^50^ Given the number of SLC transporters known to be expressed within the GI tract, it is conceivable that the accumulation of anthracyclines relies on the activity of 1 or more transporters serving a secretory function at the basolateral membrane of intestinal enterocytes, such as OCT1,^253^ to undergo intestinal secretion and thereby induce injury.

### D Vinca alkaloids

Vinca alkaloids were among the first cancer drugs discovered in the 20^th^ century, and they are derived from the Madagascar periwinkle (*Catharanthhus roseus*).^254^ Vincristine is a cornerstone treatment of various cancers, including acute lymphoblastic leukemia, lymphomas, and sarcomas. The related alkaloids vinorelbine, vinblastine, vindesine, and vinflunine are also widely employed across various tumor types, either alone or in combination with other modalities.^113^^,^^116^^,^^255^ These compounds primarily function by disrupting microtubule dynamics, which are fundamental to cellular proliferation.^256^ This generally involves the binding with microtubules at the *β*-tubulin site, which halts microtubule polymerization.^113^^,^^117^ This impedes mitotic spindle genesis, leading to cell cycle arrest and apoptosis. The unwanted toxicities of Vinca alkaloids include peripheral neurotoxicity due to neuronal cytoskeleton alterations and axon degradation,^257^^,^^258^ GI irritation resulting from epithelial cell damage that affects intestinal transit and gastric emptying,^259^ hair loss,^260^ and myelosuppression. The prevalence of these toxicities differs slightly among Vinca alkaloid agents,^261^ and peripheral neurotoxicity is considered to be the most notable adverse effect that leads to dose interruptions and discontinuations.^262^ This, in turn, may negatively impact therapeutic outcome.^263^, ^264^, ^265^, ^266^ Reported interactions between Vinca alkaloids and SLC transporters are summarized in Table 1.

#### 1 Vincristine-induced peripheral neuropathy

Vincristine-induced peripheral neuropathy (VIPN) ranges from mild to severe and is exceedingly difficult to predict and prevent.^267^ The exact intracellular mechanism of pathogenesis of VIPN development is still under investigation but has been shown preclinically to be NLR family pyrin-containing domain 3 inflammasome–dependent.^268^ Given that the NLR family pyrin-containing domain 3 complex is assembled and localized intracellularly,^269^ it is hypothesized that the initiation and severity of VIPN is tied to intracellular accumulation of vincristine in immune cells and in peripheral nerves via SLC-dependent transport. Indeed, there is evidence of direct damage to DRG nerves by vincristine.^270^

Vincristine was shown in vitro to be taken up by cells expressing members of the OATP family, specifically OATP1B1, OATP1B3, and OATP2B1.^271^ The uptake of vincristine into suspended rat hepatocytes was then shown to be modulated by inhibitors of these transporters, as well as inhibitors of efflux transporters. However, the effect of OATP inhibition in suspended human hepatocytes was far less pronounced. In a separate study, vincristine transport was screened in HeLa and HEK293 cells overexpressing a wide range of xenobiotic transporters, with OATP1B3 and the orthologous murine transporter Oatp1b2 showing the highest uptake efficiency.^114^ These transporters are expressed in human and mouse DRG, respectively. Importantly, Oatp1b2 deficiency in mice was shown to be protective against VIPN, supporting the notion that OATP-driven transport of vincristine is the initiating event that allows VIPN to develop. This was paired with the observation that plasma concentrations of vincristine were not altered by Oatp1b2 deficiency in mice, although the accumulation of vincristine in the DRG was reduced compared with wild-type mice. Nilotinib was used in this study as a pharmacological inhibitor of Oatp1b2, and pretreatment with this agent was found to offer protection from VIPN. Dipyridamole, another agent capable of inhibiting OATP-type transporters, has been reported to reduce intracellular vincristine in hiPSC-derived neurons and protect from VIPN in mice, further substantiating the role of OATP-mediated transport in the etiology of VIPN.^272^^,^^273^

Clinical trials have been performed to identify agents capable of ameliorating VIPN, including 1 in which orally administered curcumin was observed to significantly reduce the occurrence of VIPN in a population of pediatric patients with acute lymphoblastic leukemia.^274^ This evidence, in conjunction with reports that curcumin interacts with OATP-type transporters, suggests that inhibition of this mechanism may be responsible for the observed changes to the pathogenesis of VIPN.^275^^,^^276^ Efforts have also been made to identify genetic variants of transporters and metabolizing enzymes relevant to the handling of vincristine, albeit with modest success. For example, SNPs in the gene encoding for multidrug resistance-associated protein 2 (*ABCC2*) were significantly associated with the development of VIPN, although the underlying mechanism for this protective effect is not entirely clear.^277^^,^^278^ Moreover, it is unknown whether these variants are associated with gain or loss of function. Although historically much emphasis has been placed on a connection of chemotherapy-induced peripheral neuropathy with ABC transporter variants, the role of SLCs as mediators of VIPN or other toxicities induced by Vinca alkaloids is still in its infancy. It is anticipated that future studies are needed to clearly define the utility of SLC-mediated initiation as an intervention strategy to prevent toxicity.

### E Taxanes

Taxanes, which include paclitaxel, docetaxel, and cabazitaxel, are antineoplastic agents that have revolutionized the treatment landscape for a variety of malignancies. Paclitaxel, the inaugural member of this class, was first isolated in 1971 from the bark of *Taxus brevifolia* during a National Cancer Institute screening program for antineoplastic natural products.^279^ Despite its promise as a cytotoxic agent, early development was hindered by poor aqueous solubility and limited natural availability.^279^^,^^280^ These barriers stalled its development until the advent of its semisynthetic production in 1988,^281^ reigniting interest in its therapeutic potential. It was the seminal work of Susan B. Horwitz, which demonstrated their ability to promote and stabilize microtubules, ultimately leading to mitotic arrest and cell death, which further propelled its clinical advancement.^282^ Taxane-based therapies have been widely adopted and approved for use among naive, relapsed, or refractory cancers, including breast, prostate, bladder, head and neck, gastric, and non–small-cell lung cancers.^283^, ^284^, ^285^, ^286^ The efficacy of taxanes arises from their ability to target rapidly dividing cancer cells through microtubule polymer stabilization that disrupts the cell cycle, alters apoptosis, and impairs angiogenesis.^282^ To date, more than 5000 clinical trials have been conducted involving paclitaxel according to the National Institute of Health clinical trials database, with >3000 using the semisynthetic derivatives docetaxel and cabazitaxel (clinicaltrials.gov).

The therapeutic use of taxanes is frequently accompanied by adverse effects, most notably nausea, vomiting, alopecia, myelosuppression (neutropenia and leukopenia), peripheral neurotoxicity, and cognitive impairment.^287^^,^^288^ Recent evidence points to an emerging role for SLC transporters in modulating these events by governing drug uptake in tissues, thereby contributing to toxicities such as peripheral neurotoxicity and myelosuppression. Moreover, interindividual variability in SLC transporter expression and function, potentially driven by genetic polymorphisms, could help explain the heterogeneous responses observed in patients undergoing taxane-based therapy.

#### 1 Paclitaxel-induced peripheral neuropathy

Paclitaxel-induced peripheral neurotoxicity (PIPN) is a dose-limiting toxicity that presents as sensory disturbances, including numbness, paresthesia, heightened sensitivity to cold and touch, burning pain, and tingling, typically manifesting in a glove-and-stocking distribution.^289^ Remarkably, PIPN has been reported to occur in ∼97% of patients,^290^ with symptoms emerging within the first 24 hours after treatment. Among those acutely affected, 60%–70% develop chronic, often irreversible neuropathy, persisting for years or even decades after cessation of treatment.^290^ Although the exact mechanism for PIPN is not completely understood, current evidence suggests two primary pathological processes, namely (1) indirect neuronal damage driven by activated immune cells and neuroinflammatory responses and (2) direct injury to the DRG, which is considered a primary site of accumulation and toxicity within the peripheral nervous system.^291^^,^^292^ Recent preclinical studies have highlighted the promise of targeting the OATP1B family of SLCs to mitigate PIPN. Although OATP transporters are expressed primarily on the basolateral membrane of hepatocytes,^293^^,^^294^ they were more recently found in tissues of the central and peripheral nervous system.^295^ Their role in the pharmacokinetic profile of taxanes has been well-documented in reports showing that altered OATP1B activity, whether due to genetic polymorphisms, drug-drug interactions, or disease states, can significantly impact drug exposure, efficacy, and toxicity.^296^^,^^297^ Understanding the role of OATP1B transporters in the disposition of taxanes is therefore critical for optimizing dosing strategies, predicting interpatient variability, and improving therapeutic efficacy while minimizing adverse effects.

The hypothesis that SLC transporters contribute to taxane-induced toxicity in DRG neurons is supported by several key observations. First, paclitaxel readily accesses the DRG compartment and long persists after its elimination from plasma.^298^^,^^299^ Second, taxanes, specifically paclitaxel and docetaxel, are established substrates of OATP1B family members, particularly OATP1B1 and OATP1B3 in humans, as well as the murine and rat ortholog Oatp1b2.^118^^,^^120^^,^^297^^,^^300^, ^301^, ^302^, ^303^, ^304^, ^305^ Third, transcript and protein expressions of OATP1B transporters have been confirmed in DRG tissues from animals and humans.^114^^,^^306^ Studies have demonstrated that taxanes accumulate intracellularly via facilitated transport by the OATP family members, and moreover, paclitaxel itself acts as a potent inhibitor of both OATP1B1- and OAT1B3-mediated transport.^119^^,^^121^^,^^307^, ^308^, ^309^, ^310^, ^311^, ^312^, ^313^, ^314^, ^315^

The role of Oatp1b2-mediated transport of paclitaxel as an initiating event in PIPN was first reported in mice by comparing the severity of mechanical allodynia in wild-type mice to animals genetically deficient in Oatb1b2 after paclitaxel treatment.^306^ Oatp1b2-deficient mice were protected from PIPN and showed reduced paclitaxel concentrations within their DRG. Furthermore, the observed protection was not attributed to any concurrent changes in the plasma pharmacokinetics of paclitaxel, as measures of systemic exposure were similar between mouse genotypes. Additional studies have confirmed these pharmacokinetic findings, reporting only relatively modest systemic exposure differences in paclitaxel or docetaxel between wild-type mice and Oatp1b2-deficient animals.^120^^,^^301^^,^^316^ Follow-up studies with nilotinib, used as a pharmacological inhibitor of Oatp1b2, verified that pharmacological blockade could recapitulate the protective phenotype observed in knock-out animals. Importantly, the translational success of this preventative strategy is dependent on the inhibitor not impeding the antitumor activity of paclitaxel. Preliminary in vitro and in vivo studies in cell lines and xenograft models of breast cancer demonstrate the absence of antagonistic effects of nilotinib on paclitaxel activity. This proof-of-concept finding has provided the impetus for an ongoing phase1b clinical trial (NCT04205903) aimed at evaluating OATP1B inhibition as a strategy to prevent PIPN in patients with breast cancer. Furthermore, the combination of nilotinib to paclitaxel as a combination strategy is being evaluated as a separate therapeutic approach in refractory solid tumors (NCT02379416), and although improvement in neuropathy was not an endpoint for the study, published results from this phase 1 study demonstrated that patients treated with the nilotinib-paclitaxel combination had a lower incidence and severity of neuropathy compared with historical data with other paclitaxel-based therapy.^317^ These preliminary findings support the thesis that inhibition of OATP1B transporters in patients is generally safe, does not alter the pharmacokinetic profile of paclitaxel, and could be an effective neuroprotective strategy for PIPN. As such, randomized, placebo-controlled, confirmatory phase 2 studies are urgently needed to verify this concept. Independent lines of evidence further support a role of OATP1B transporters in mediating taxane-induced neurotoxicity. For example, clinical data comparing taxane-containing regimens for the treatment of castration-resistant prostate cancer have revealed that cabazitaxel, a weak substrate for OATP1B transporters, is associated with a significantly lower incidence of neuropathy compared with docetaxel.^26^^,^^120^^,^^318^

In addition, glycyrrhizic acid has been shown to inhibit Oatp1a4- and Oatp1b2-mediated uptake of paclitaxel in rodent F11 neuronal cells, leading to reduced cytotoxicity in vitro. It should be noted that in mice, Oatp1a4 exhibits a partially overlapping substrate specificity with Oatp1b2, including that of paclitaxel, suggesting that Oatp1a4 may be partially responsible for the neuronal transport of paclitaxel and the development of PIPN. Redundancy in transport is not uncommon, and inhibition of both transporters by a single agent may afford a potent protective strategy. Translating these findings in vivo, mice cotreated with glycyrrhizic acid and paclitaxel displayed no significant changes in behavioral or electrophysiological markers of neuropathy compared with negative controls, in stark contrast to mice treated with paclitaxel alone.^319^ This preclinical evidence supports the potential clinical utility of glycyrrhizic acid as a neuroprotective agent. Beyond glycyrrhizic acid, an initial phase 2 clinical trial demonstrated that vitamin E supplementation can reduce the incidence of PIPN.^320^ However, in a randomized phase 3 trial, there was no beneficial effect of high-dose vitamin E on PIPN.^321^ Interestingly, vitamin E was identified as a DRG-specific biomarker under genetic or pharmacologic perturbations of Oatp1b2.^114^ Collectively, these studies underscore the pivotal role of OATP1B transporters, particularly Oatp1b2, in mediating the accumulation of taxanes in DRG and the subsequent development of PIPN. This provides a compelling rationale for further exploration of transporter-targeted interventions as a means to improve the therapeutic index of taxane-based chemotherapy.

#### 2 Taxane-induced cognitive impairment

In addition to peripheral neurotoxicity, taxanes can induce both short- and long-term effects on the central nervous system,^322^ demonstrated by reports of ataxia, emotional distress, and cognitive impairments in patients. This supports the notion that these agents may directly injure the brain and cause taxane-induced cognitive impairment (TICI). Drug transporters facilitate the movement of drugs across the cell membrane. However, crossing the blood-brain barrier (BBB) presents a unique set of challenges.^323^ Extensive work has been done to characterize the role of efflux transporters on the role of TICI, notably P-glycoprotein (ABCB1),^324^ based on the thesis that this transporter is primarily responsible for the efflux of taxanes from the central nervous system, and that inhibition of this mechanism leads to increased brain penetration of taxanes. One report shows that paclitaxel reaches concentrations greater than 200 nM within the hippocampus,^325^ an area of the brain important for learning and memory. This may indicate that enrichment of taxanes within specific regions of the brain may contribute to TICI, like the hippocampus, amygdala, and cerebellum, but not the cortex.

To date, no study has directly demonstrated a relationship between TICI- and SLC-dependent transport. However, it is known that OATP1A2 and OATP2B1 are localized to the BBB in human tissues.^326^^,^^327^ It is also known that paclitaxel is a substrate for OATP1A2 but not for OATP2B1.^121^^,^^306^ Nonetheless, the presence of at least a single transporter that can translocate paclitaxel across the BBB supports the hypothesis that its initial entry into the brain may be mediated, at least in part, by an SLC transporter. Beyond the BBB, there is evidence that several OATP-type transporters are detectable in a variety of regions within the central nervous system.^328^ With ample evidence to show the contribution of ABC transporters to the brain accumulation of taxanes and the development of TICI, the availability of additional evidence favoring an SLC-mediated accumulation mechanism may improve our understanding of this phenomenon and allow for improved, targeted preventative measures.

#### 3 Taxane-induced myelosuppression

Among the adverse effects of taxanes, myelosuppression appears to have the least direct evidence for an involvement of SLC transporters. Instead, current hypotheses largely implicate expression of ABC transporters in the bone marrow, particularly P-glycoprotein, in preventing this toxicity, a notion that parallels findings reported previously for the P-glycoprotein substrate vincristine.^17^ For instance, cabazitaxel produces the most severe myelosuppressive effects among the available taxanes, likely because it is a weaker substrate for P-glycoprotein compared with paclitaxel and docetaxel.^329^ Interestingly, pharmacogenomic studies have identified a SNP in *SLCO1B3* (rs11045585, a decrease-of-function variant) that is associated with an increased risk of docetaxel-induced myelosuppression.^330^^,^^331^ However, it remains unclear whether this variant is causally linked with elevated plasma concentrations of docetaxel due to an elimination defect associated with impaired hepatic uptake, suggesting a quasi–dose-dependent toxicity due to reduced clearance or if it alters intracellular drug concentrations in specific cell types, such as bone marrow progenitors. The current evidence favors the former scenario, implying that decreased clearance and subsequent higher systemic exposure are key contributors to the observed toxicity. Beyond this genetic correlation, there is limited evidence linking taxane-induced myelosuppression directly to SLC transporter activity, and further investigation is required to shed light on this mechanism.

### F Tyrosine kinase inhibitors

Tyrosine kinase inhibitors (TKIs) developed over the past 25 years have radically altered the landscape of cancer treatment. Imatinib was the first TKI to be approved for human use near the turn of the millennium to treat Philadelphia chromosome-positive chronic myeloid leukemia as an agent targeting breakpoint-cluster region–Abelson (associated with Philadelphia chromosome).^4^^,^^332^ Since then, more than 80 agents in this class have been approved that target a diverse array of kinases, and that number is expected to further increase.^333^ Although not unique to the class of TKIs, the oral mode of dosing lends especially great risk for extensive interindividual pharmacokinetic variability, which can lead to increased toxicity or diminished efficacy despite their purported wide therapeutic window. This variability can arise due to unintentional drug-drug interactions in a real-world, polypharmacy setting^334^, ^335^, ^336^ but can be further influenced by intrinsic effects such as organ impairment and differential expression of transporters and enzymes.^337^ Clinically, the use of TKIs has been associated with severe adverse events in a significant proportion of patients.^338^ These include cardiotoxicity from TKIs such as osimertinib, crizotinib, and the breakpoint-cluster region–Abelson (associated with Philadelphia chromosome)–targeting agents nilotinib, dasatinib, and ponatinib^339^, ^340^, ^341^; hepatotoxicity from TKIs such as pazopanib, sunitinib, and erlotinib^342^, ^343^, ^344^, ^345^; skin, rash, and hand-foot skin reactions from erlotinib, gefitinib, sorafenib, and sunitinib^346^^,^^347^; and ocular toxicity with the use of afatinib, crizotinib, and dasatinib.^348^ The occurrence of these toxic events can often be partially attributed not only to on-target effects but also to unexpected variability in systemic drug exposure and subsequent distribution to sites of injury associated with drug-drug interactions.^36^^,^^349^, ^350^, ^351^, ^352^ That being said, the class of TKIs possesses much diversity, and that diversity is reflected in the varied and difficult to predict toxicities that arise with their use.^353^

TKIs and their interaction with the transportome have been the subject of intense investigation, and several TKIs have been identified as substrates of xenobiotic transporters, including OCTs, OATs, and OATPs.^36^^,^^354^, ^355^, ^356^, ^357^, ^358^ The ability of many TKIs to inhibit transporters is also of potential therapeutic significance, and their inhibitory properties often involve noncompetitive mechanisms that are dependent on the inhibition of kinases involved in the activation of SLCs by phosphorylation.^114^^,^^359^, ^360^, ^361^, ^362^, ^363^ The extent to which post-translational regulation of SLC transporters by a kinase that is sensitive to inhibition by TKIs contributes to clinically relevant drug-drug interactions is under further investigation. Table 2^364^, ^365^, ^366^, ^367^, ^368^, ^369^, ^370^, ^371^, ^372^, ^373^, ^374^, ^375^, ^376^, ^377^, ^378^, ^379^, ^380^, ^381^, ^382^, ^383^, ^384^, ^385^, ^386^, ^387^, ^388^, ^389^, ^390^, ^391^, ^392^, ^393^, ^394^, ^395^, ^396^, ^397^, ^398^, ^399^, ^400^, ^401^, ^402^, ^403^, ^404^, ^405^, ^406^, ^407^, ^408^, ^409^, ^410^, ^411^, ^412^, ^413^, ^414^, ^415^, ^416^, ^417^, ^418^, ^419^, ^420^, ^421^, ^422^, ^423^, ^424^, ^425^, ^426^, ^427^, ^428^, ^429^, ^430^, ^431^ summarizes the known TKI toxicities and interactions with SLC transporters. Despite having been the focus of intense research, experimental challenges and knowledge gaps with this drug class remain,^432^^,^^433^ and there are many inconsistencies in the published literature on the role of specific SLCs in the transport of specific TKIs.^4^^,^^293^^,^^434^

**Table 2:** Toxicities and SLC interactions of tyrosine kinase inhibitors

Drug	Toxicity^a^	Substrate^b^	Inhibitor^c^	References
Acalabrutinib	Dermal toxicity Gastrointestinal	--	OATP1B3	^360^^,^^364^
Afatinib	Dermal toxicity Gastrointestinal	--	MATE1, OATP1B1, and OATP1B3	^293^^,^^360^^,^^365^^,^^366^
Alectinib	Cardiotoxicity Dermal toxicity Gastrointestinal Hepatotoxicity Nephrotoxicity	--	--	^367^^,^^368^
Asciminib	Cardiotoxicity Myelosuppression	--	OATP1B1, OATP1B3, and OCT1	^369^
Avapritinib	Central nervous system Gastrointestinal Myelosuppression	--	MATE1 and OATP1B3	^353^^,^^360^^,^^366^
Axitinib	Dermal toxicity Gastrointestinal	OATP1B1 and OATP1B3	GLUT1, MATE1, and OATP1B3	^360^^,^^366^^,^^370^^,^^371^
Binimetinib	Cardiotoxicity Gastrointestinal	OATP1B1	OATP1B1 and OATP1B3	^43^^,^^293^^,^^372^^,^^373^
Bosutinib	Cardiotoxicity Hepatotoxicity Gastrointestinal	--	GLUT1, MATE1, OATP1B3, and OCT2	^153^^,^^360^^,^^366^^,^^371^^,^^374^^,^^375^
Brigatinib	Hepatotoxicity Gastrointestinal	OATP1B1	MATE1 and OATP1B3	^43^^,^^360^^,^^366^
Cabozantinib	Dermal toxicity Gastrointestinal	OATP1B1	MATE1 and OATP1B3	^43^^,^^360^^,^^366^
Capmatinib	Gastrointestinal Hepatotoxicity Myelosuppression Ocular toxicity	--	MATE1 and OATP1B1	^366^^,^^376^, ^377^, ^378^
Ceritinib	Cardiotoxicity Gastrointestinal Hepatotoxicity Ocular toxicity	OATP1B1	MATE1 and OATP1B3	^43^^,^^360^^,^^366^
Cobimetinib	Dermal toxicity Gastrointestinal Hepatotoxicity	OATP1B1	MATE1, OATP1B1, and OATP1B3	^43^^,^^293^^,^^360^^,^^366^
Crizotinib	Cardiotoxicity Dermal toxicity Hepatotoxicity Myelosuppression Ocular toxicity	OATP1B1, OCT1, OCT2, and OCT3	MATE1 and OATP1B3	^43^^,^^339^^,^^346^^,^^360^^,^^366^
Dabrafenib	Dermal toxicity Gastrointestinal	OATP1B1	MATE1, OATP1B1, and OATP1B3	^43^^,^^293^^,^^360^^,^^366^^,^^379^
Dacomitinib	Dermal toxicity Gastrointestinal	--	MATE1 and OATP1B3	^360^^,^^366^^,^^380^
Dasatinib	Cardiotoxicity Gastrointestinal Myelosuppression Ocular toxicity	OATP1B1, OATP1B3, OCT1, OCT2, and OCT3	MATE1, MATE2K, OATP1B3, OCT1, OCT2, and OCT3	^43^^,^^357^^,^^360^^,^^366^^,^^381^, ^382^, ^383^
Encorafenib	Cardiotoxicity Dermal toxicity Gastrointestinal Ocular toxicity	OATP1B1	OATP1B1 and OATP1B3	^43^^,^^293^^,^^360^^,^^372^^,^^373^
Entrectinib	Central nervous system Gastrointestinal	--	MATE1 and OATP1B3	^360^^,^^366^^,^^384^
Erdafitinib	Dermal toxicity Ocular toxicity	OATP1B1	MATE1 and OATP1B3	^43^^,^^360^^,^^366^^,^^385^
Erlotinib	Dermal toxicity Gastrointestinal	OAT3, OATP2B1, and OCT2	MATE1, MATE2K, OCT1, OCT2, and OCT3	^365^^,^^366^^,^^381^^,^^386^^,^^387^
Fedratinib	Encephalopathy Gastrointestinal Myelosuppression	--	MATE1 and OATP1B3	^360^^,^^366^^,^^388^^,^^389^
Futibatinib	Dermal toxicity Hepatotoxicity Ocular toxicity	OATP1B1	--	^43^^,^^390^^,^^391^
Gefitinib	Dermal toxicity	OATP1B3, OCT1, OCT2, and OCT3	GLUT1, MATE1, MATE2K, OATP1B3, OCT1, and OCT3	^357^^,^^360^^,^^365^^,^^366^^,^^371^^,^^380^, ^381^, ^382^
Gilteritinib	Gastrointestinal Myelosuppression	OATP1B1	MATE1, OATP1B1, and OATP1B3	^43^^,^^293^^,^^360^^,^^366^^,^^392^^,^^393^
Ibrutinib	Cardiotoxicity Dermal toxicity Ocular toxicity	--	MATE1 and OATP1B3	^360^^,^^364^^,^^366^^,^^394^
Imatinib	Dermal toxicity Gastrointestinal Myelosuppression Nephrotoxicity	OATP1A2, OCT1, and OCTN2	GLUT1, MATE1, MATE2K, OATP1B3, OCT1, OCT2, and OCT3	^360^^,^^366^^,^^371^^,^^374^^,^^381^^,^^395^, ^396^, ^397^
Lapatinib	Cardiotoxicity Dermal toxicity Gastrointestinal	OATP1B1	OATP1B1 and OATP1B3	^43^^,^^293^^,^^360^^,^^398^^,^^399^
Larotrectinib	Dermal toxicity Gastrointestinal Hepatotoxicity	OATP1B1	--	^43^^,^^400^
Lenvatinib	Cardiotoxicity Dermal toxicity Gastrointestinal	--	MATE1, OATP1B1, and OATP1B3	^293^^,^^360^^,^^366^^,^^401^
Lorlatinib	Cardiotoxicity Central nervous system Peripheral neuropathy	--	MATE1, OATP1B1, and OATP1B3	^293^^,^^366^^,^^368^^,^^402^
Midostaurin	Gastrointestinal Myelosuppression	OATP1B1	MATE1 and OATP1B1	^43^^,^^293^^,^^360^^,^^403^
Mobocertinib	Cardiotoxicity Dermal toxicity Nephrotoxicity Ocular toxicity	--	--	^404^
Neratinib	Dermal toxicity Gastrointestinal	--	MATE1 and OATP1B3	^360^^,^^366^^,^^405^
Nilotinib	Cardiotoxicity Dermal toxicity Gastrointestinal	OATP1B1 and OATP1B3	GLUT1, MATE1, MATE2K, OAT3, OATP1B3, OCT1, OCT2, and OCT3	^153^^,^^360^^,^^366^^,^^371^^,^^381^^,^^382^^,^^406^, ^407^, ^408^
Nintedanib	Gastrointestinal Hepatotoxicity	OATP1B1	MATE1 and OATP1B3	^43^^,^^360^^,^^366^^,^^409^
Osimertinib	Cardiotoxicity Dermal toxicity Hepatotoxicity	OATP1B1	MATE1, OATP1B1, and OATP1B3	^43^^,^^293^^,^^360^^,^^365^^,^^366^^,^^410^^,^^411^
Pacritinib	Cardiotoxicity Dermal toxicity Gastrointestinal Myelosuppression	--	OCT1 and OCT2	^412^
Pazopanib	Cardiotoxicity Dermal toxicity Hepatotoxicity	OATP1B1 and OCT1	GLUT1, MATE1, OATP1B1, OATP1B3, and OCT2	^43^^,^^153^^,^^293^^,^^360^^,^^366^^,^^371^^,^^413^, ^414^, ^415^
Pemigatinib	Dermal toxicity Gastrointestinal	--	MATE1	^360^^,^^435^
Pexidartinib	Hepatotoxicity Neuropathy	OATP1B1	MATE1	^43^^,^^366^^,^^416^^,^^417^
Ponatinib	Cardiotoxicity Hepatotoxicity	--	MATE1 and OATP1B3	^340^^,^^360^^,^^366^^,^^375^^,^^418^
Pralsetinib	Cardiotoxicity Gastrointestinal Hepatotoxicity	OATP1B1	MATE1 and OATP1B1	^43^^,^^366^^,^^376^^,^^419^^,^^420^
Regorafenib	Dermal toxicity Gastrointestinal	OATP1B1	MATE1	^43^^,^^366^^,^^421^
Ripretinib	Cardiotoxicity Dermal toxicity Gastrointestinal	--	MATE1 and OATP1B1	^366^^,^^376^^,^^422^^,^^423^
Ruxolitinib	Myelosuppression	OATP1B1	MATE1	^43^^,^^366^^,^^424^
Selumetinib	Cardiotoxicity Dermal toxicity Gastrointestinal Ocular toxicity	OATP1B1		^43^^,^^425^
Sorafenib	Dermal toxicity Gastrointestinal Hepatotoxicity	OATP1B1, OATP1B3, OAT6, and OCT1	MATE1	^346^^,^^366^^,^^426^, ^427^, ^428^
Sunitinib	Dermal toxicity Gastrointestinal Myelosuppression	OATP1B1, OCT1, OCT2, and OCT3	MATE1, MATE2K, OCT1, OCT2, and OCT3	^43^^,^^357^^,^^366^^,^^381^
Tepotinib	Gastrointestinal Hepatotoxicity	--	--	^429^
Tivozanib	Cardiotoxicity Gastrointestinal	--	OATP1B1	^376^
Trametinib	Dermal toxicity Gastrointestinal Ocular toxicity	--	MATE1, OATP1B1, and OATP1B3	^293^^,^^360^^,^^366^^,^^379^
Tucatinib	Dermal toxicity Gastrointestinal	OATP1B1	MATE1 and OATP1B1	^43^^,^^366^^,^^376^^,^^430^
Vandetanib	Cardiotoxicity Dermal toxicity Gastrointestinal	OATP1B1 and OATP1B3	MATE1 and OCT2	^153^^,^^366^^,^^382^^,^^431^
Zanubrutinib	Cardiotoxicity Dermal toxicity Gastrointestinal Myelosuppression	--	OATP1B1	^364^^,^^376^

a Listed toxicities were identified based on the FDA insert information, alphabetically.

b Interaction of tyrosine kinase inhibitors with SLCs as substrates.

c Interaction of tyrosine kinase inhibitors with SLCs as inhibitors.

#### 1 Tyrosine kinase inhibitor-induced hepatotoxicity

Hepatic injury is a commonly occurring side effect in patients receiving TKI-based therapy.^343^^,^^350^ Since the majority of TKIs undergo metabolism by cytochrome P450 (CYP) enzymes in the liver, both parent drugs and toxic metabolites have been implicated in the initiation of hepatotoxicity.^436^ Polymorphisms in *SLCO1B1* have been associated with altered risk of sorafenib-related toxicities in a cohort of patients.^426^ There is some evidence to suggest a role of OCT1 in the hepatic transport of sorafenib.^427^ However, this could not be confirmed in other investigations, and thus, the clinical implications of this relationship remain obscure.^433^^,^^437^

The uptake of pazopanib, a TKI that contains a black box warning for hepatotoxicity in the label, into hepatocytes in mice was recently found to be dependent on transporter-mediated processes.^43^ In an agnostic TKI screen to identify OATP1B1 substrates, pazopanib was identified as a previously recognized substrate. This finding was confirmed in silico molecular modeling of cryo-electron microscopy structures of human OATP1B1 to verify the substrate interactions of pazopanib. The role of OATP1B1 on pazopanib’s main kinase target, vascular endothelial growth factor receptor, was reduced in isolated hepatocytes from mice lacking all Oatp1a and Oatp1b isoforms. Furthermore, the liver-to-plasma ratios of pazopanib in Oatp1a/1b-deficient mice were reduced significantly without elevated levels of hepatic injury markers aspartate aminotransferase and alanine aminotransferase. Additionally, these phenotypes were not attributed to differences in pazopanib pharmacokinetics among the genotypes. In a separate study, pazopanib uptake in isolated murine and human hepatocytes was sensitive to OCT1 inhibition. In direct uptake experiments, pazopanib was a substrate for OCT1 and less so for OATP1B1. The assertion that pazopanib is a substrate for OCT1 provides evidence for the involvement of multiple transporters in the hepatic disposition of this drug. The comparison of kinase activity in isolated hepatocytes of Oatp1a/1b-deficient mice and wild-type mice supports this: exposure to 10 μM pazopanib reduced vascular endothelial growth factor receptor 2 kinase activity by ∼90% in wild-type hepatocytes and by ∼30% in Oatp1a/1b-deficient hepatocytes. OCT1-mediated uptake of pazopanib can explain this residual inhibition.

In a meta-analysis of studies with hepatotoxic TKIs that target the vascular endothelial growth factor receptor, the occurrence of elevated serum markers of liver injury varied by study.^370^ Potent perturbations of OATP1B1-mediated transport may confer substantial risk to indirect toxicities and hypersensitivities due to impaired hepatic handling of xenobiotic substrates or the detoxification of endogenous toxins. For example, homozygous null mutations in OATP1B1 and OATP1B3 in subjects with rotor syndrome is associated with hyperbilirubinemia as a consequence of reduced hepatic handling (uptake and excretion) of bilirubin.^438^ As such, several TKIs that potently inhibit OATP1B1 and OATP1B3 transport activity, such as nilotinib, have been linked to clinical elevations of bilirubin in the absence of liver injury.^360^^,^^376^^,^^439^^,^^440^ Therefore, it is important to highlight that TKIs themselves can elevate serum bilirubin in a manner that is potentially attributable to inhibition of an SLC transporter.^441^

#### 2 Tyrosine kinase inhibitor-induced skin toxicity

Adverse effects related to the skin are detrimental to the quality of life of patients receiving TKIs and are especially prevalent in patients receiving epidermal growth factor receptor (EGFR) inhibitors, although these effects are reported with the use of a diverse array of TKIs.^364^^,^^442^^,^^443^ Using an siRNA library screen, the transporter OAT6 (SLC22A20) was identified as an uptake transporter of sorafenib in human keratinocytes.^346^ In mice, sorafenib keratinocyte toxicity was attenuated by cotreatment with the OAT6 inhibitor probenecid, without antagonizing the efficacy of sorafenib against cancer cells. Interestingly, sorafenib concentrations in skin biopsies in humans were decreased by pretreatment with probenecid, although this was possibly a consequence of changes in the metabolic ratio of sorafenib-glucuronide to parent drug and in circulating parent drug levels in the presence of probenecid.^444^ Since probenecid seems not to be an ideal agent to introduce to sorafenib treatment, an alternative OAT6 inhibitor would be of interest. Given the burden these adverse events pose, and given the need for better ways to address them,^445^ more thorough investigation of this phenomenon is warranted.

#### 3 Tyrosine kinase inhibitor-induced pseudoinjury

The disruption of tissue homeostasis, mediated by the disruption of normal SLC function, can starve tissues of essential nutrients. This is in addition to the effect of TKIs on OATP transporters that can affect the handling of endogenous bilirubin, as described above. This can be a global phenomenon in regulating tissue homeostasis throughout the body. For instance, crizotinib can reduce blood glucose levels independently of insulin and glucagon and cause cardiac glucose transporter (GLUT)4 upregulation in diabetic rats.^446^ Another report indicates that a panel of TKIs, including nilotinib, imatinib, gefitinib, and pazopanib, can bind directly to GLUT1, as well as inhibit the transport of 2-deoxyglucose in cell-based models.^371^ Thus, the cardiotoxic effects associated with the use of TKIs, in addition to their pleiotropic effects on kinases, can also be explained by their effects on transporters essential to regulating cardiac homeostasis.^339^^,^^340^^,^^369^^,^^447^

Beyond interference by TKIs of OATP1B1-mediated transport of bilirubin, a disruption of SLC function can influence other circulating markers that are clinically used to identify organ toxicity. For example, elevations in serum creatinine, used to calculate glomerular filtration rate and assess kidney function, are observed with the use of agents like sunitinib.^448^^,^^449^ Although sunitinib has been shown to be toxic to HK-2 renal tubular epithelial cells, it is also known to inhibit OCT2, MATE1, and MATE2-K.^381^ These comprise the SLC cation transporters responsible for the active secretion from blood into renal tubular cells and the terminal excretion of serum creatinine from those cells, respectively.^381^^,^^450^^,^^451^ This pathway is responsible for some fraction of creatinine clearance between 10% and 40%, although this varies in literature.^452^^,^^453^ As such, it can become difficult to disentangle the role of direct toxicity or SLC transporter inhibition in serum creatinine elevation. Several TKIs have the ability to potently inhibit these organic cation transporters. This interaction can yield a “pseudoinjury,” where serum creatinine is elevated in some cases without direct evidence that the kidney is actually injured.^377^^,^^453^^,^^454^ Similar clinical observations have been made with OCT2 inhibitors that do not belong to the class of TKIs, such as pantoprazole.^455^^,^^456^ These issues can become difficult to disentangle in clinical practice. For example, a recent case study reported a grade 3 adverse reaction with the use of the CDK4/6 inhibitor ribociclib, a known OCT2 inhibitor, as indicated by elevated serum creatinine with a few other abnormalities.^457^ Serum creatinine levels returned to normal values after cessation of therapy with ribociclib and then elevated again upon reinitiation of therapy. Given that kidney injury of this severity with ribociclib is rare, this supports the thesis that alternate circulating biomarkers should be considered to detect the existence of kidney injury and exclude a pseudoinjury caused by transient, SLC-mediated phenomena, especially with agents that are suspected inhibitors of OCT2 and MATE1/MATE2K. Beyond the multiple instances of pseudoinjury, several TKIs, including axitinib and sorafenib, are actually capable of inducing high-grade kidney-related adverse events, according to a meta-analysis,^458^ although the rate of nephrotoxic events varies greatly by study and by TKI. Given the reliance on serum creatinine to detect kidney injury in patients, alternative serum markers such as cystatin C, which is not transported by OCT2 and MATE1/MATE2K, should be used in conjunction with serum creatinine to distinguish kidney injury from inhibition of creatinine transporters.

### G Small-molecule payloads of antibody-drug conjugates

Over the past decade, ADCs have emerged as a class of transformative therapies in both solid and hematological malignancies.^459^, ^460^, ^461^ The unique composition of an ADC combines a monoclonal antibody linked to a small-molecule cytotoxic payload. In this way, they selectively deliver potent small-molecule payloads to the desired site of action while minimizing off-target adverse effects.^462^^,^^463^ In general, upon target binding, the ADC is internalized and trafficked to subcellular locations (eg, endosome or lysosome) from which the small molecule payload is deconjugated and released.^464^

Despite the recent approvals and ongoing clinical development, the effectiveness of ADCs has been limited by the onset of dose-limiting toxicities. Although the overarching development objective of ADCs is to widen the relatively narrow therapeutic window of these potent cytotoxins, dose-limiting toxicities remain a major determinant of patient tolerability, dose modifications, discontinuations, and patient quality of life.^465^, ^466^, ^467^ The initiating mechanisms by which these agents induce dose-limiting toxicities remain to be fully elucidated. However, they have been hypothesized to be driven by off-target and nonspecific processes, including (1) target-independent uptake of the intact ADC into healthy nontargeted cells through endocytosis,^467^, ^468^, ^469^, ^470^ (2) internalization of the intact ADC via antibody binding with Fc/C-type lectin receptors,^471^, ^472^, ^473^, ^474^ or (3) the release of the small-molecule payload from either targeted or nontargeted mechanisms may cross biological membranes via a “bystander effect.”^475^ The bystander effect is described as the intentional or unintentional diffusion and cytotoxic activity in heterogenous tumor cell populations, and thus, it may contribute to the toxicity in healthy cells. Interestingly, the exposure-response relationships for key adverse events with approved ADCs suggest that increasing ADC exposure, but not exposure of the unconjugated small-molecule payload, is associated with a higher incidence and severity of these adverse events.^476^, ^477^, ^478^, ^479^, ^480^ To date, the approved ADCs contain small-molecule payloads that are classified as tubulin poisons, DNA-crosslinkers/alkylators, or topoisomerase inhibitors. Their key dose-limiting toxicities are described in Table 3. These approved ADCs target traditional pathways of cytotoxic chemotherapeutic agents. Although the expression of target antigens in specific tissues of unintended injury is generally absent or detected at very low levels,^467^^,^^477^, ^478^, ^479^, ^480^ the toxicity profiles appear to be consistent with the small-molecule payload and within a class effect (Table 3),^467^ supporting the thesis that ADC-related toxicities are primarily driven by the payload.

**Table 3:** Toxicities of antibody-drug conjugates and their payloads by mechanism class

Small-Molecule Payload	Antibody-Drug Conjugate	Target	Tumor	Toxicity^a^
Tubulin Poisons				

Monomethyl auristatin E (MMAE)	Brentuximab vedotin Enfortumab vedotin Polatuzumab vedotin Tisotumab vedotin	CD30 Nectin-4 CD79b TF	Heme Solid Heme Solid	Peripheral neuropathy Neutropenia Hepatotoxicity Ocular toxicity
Monomethyl auristatin F (MMAF)	Belantamab mafodotin	BCMA	Heme	Ocular toxicity Thrombocytopenia Hepatotoxicity
Mertansine (DM1)	Trastuzumab emtansine	HER2	Solid	Pulmonary toxicity Thrombocytopenia Hepatotoxicity Neurotoxicity Cardiotoxicity
Ravtansine (DM4)	Mirvetuximab sorfavtansine	FR*α*	Solid	Ocular toxicity Peripheral neuropathy Neutropenia

DNA-Crosslinkers				

Calicheamicin	Gemtuzumab ozogamicin Inotuzumab ozogamicin	CD33 CD22	Heme Heme	Hepatoxicity Thrombocytopenia Neutropenia
Pyrrolobenzodiazepine (PBD)	Locastuximab tesirine	CD19	Heme	Neutropenia Thrombocytopenia Pleural effusion Cutaneous toxicity

Topoisomerase Inhibitors				

SN-38	Sacituzumab govitecan	Trop-2	Solid	Neutropenia Gastrointestinal toxicity
Deruxtecan	Trastuzumab deruxtecan Datopotamab deruxtecan	HER2 Trop-2	Solid	Neutropenia Cardiotoxicity Stomatitis/mucositis Gastrointestinal toxicity

a Listed toxicities were identified based on the FDA insert information.

In support of this thesis, for example, monomethyl auristatin E (MMAE)-ADC–induced peripheral neurotoxicity has been shown to be independent of antibody target as well as cancer type (Table 3).^476^ The payload-dependent effects have been described in preclinical models where repeat administration of free MMAE can induce and recapitulate a peripheral neurotoxicity phenotype in rodents that is similar to that in humans.^475^ This observation suggests the accumulation of free MMAE may occur in the tissues of injury, such as the DRG and sciatic nerve.^481^ Based on predictions from a physiologically based pharmacokinetic model of MMAE, it is suggested that differences in tissue distribution of MMAE can be explained by differences in blood perfusion rate, tissue cell membrane penetration rate, or the involvement of presently unknown transporters.^482^ In addition, it is worth noting that polatuzumab vedotin, a CD79b-MMAE-ADC, was detected in rodents as both MMAE-ADC and CD79b-ADC, not only in highly perfused tissues, but also in tissue homogenates of the DRG and sciatic nerve,^483^ evidence that supports the nonspecific uptake and deconjugation of the ADC in these tissues. At present, MMAE-ADC targets are not known to be expressed on cells found within the peripheral nervous system in humans. Although tissue distribution has been documented for MMAE and MMAE-ADCs, it is unknown whether these observations occur with ADCs containing other tubulin poison payloads that also cause neuropathy.^484^ In further support of this supposition, it is noteworthy that systemic and local (optical) pretreatment of rodents and cynomolgus monkeys with an anti-EGFR monoclonal antibody, to saturate target engagement, did not prevent ocular injury associated with an EGFR-monomethyl auristatin F (MMAF)-ADC.^485^ Furthermore, the SN-38-ADC has an overlapping dose-limiting toxicity profile with irinotecan, a prodrug of irinotecan that causes severe GI toxicity in a manner that is partially mediated by OATP2B1-mediated uptake of SN-38 accumulation in intestinal enterocytes.^486^^,^^487^

In a retrospective analysis of data from first-in-human clinical trials with ADCs, the recommended phase 2 dose and maximum tolerated dose of an ADC largely correspond to similar doses of the small-molecule after normalization for ADC payload content (eg, dose, drug-to-antibody ratios, and molecular weight). For example, the auristatins MMAE and MMAF, synthetic derivatives of dolastatin 10, have similar small-molecule content at the maximum tolerated dose after normalization, despite completely different plasma pharmacokinetic profiles between dolastatin 10 and auristatin-based ADCs.^488^

It is estimated that only a very small fraction of an administered ADC dose can successfully reach the target tumor site.^489^ Following ADC administration, the released payload can rapidly appear within systemic circulation. In most cases with ADCs containing a cleavable linker-payload, it is anticipated that the released payload will be eliminated from the body similar to conventional clearance processes of small-molecule therapeutics, such as renal excretion, nonrenal excretion, and biotransformation. In addition, the pharmacokinetics and distribution patterns in most tissues between the released payload of a cleavable ADC and as a free small-molecule are comparable. In contrast, the released payloads from ADCs with noncleavable linkers can be molecules with different pharmacokinetic and drug disposition properties.^435^^,^^490^^,^^491^ It is also important to note that the circulating levels of the payload often exist as a linker-payload moiety; therefore, verification of payload versus linker-payload interactions with drug transporters needs to be considered. As such, drug-drug interactions with the small-molecule payload can still occur and are, at least in part, dependent on inherent distribution, metabolism, and excretion. ADCs can become victim to DDIs, and concomitant medications that have CYP3A-modifying activity pose the highest potential risk. This has warranted cautionary language in the prescribing information; however, no dose modifications due to drug-drug interactions are required for any of the approved ADCs.^492^ As such, the initiation of the aforementioned dose-limiting toxicities associated with ADCs may rely, at least in part, on the uptake of the small-molecule payload into sites of injury by one or more SLC transporters. Although transporter-dependent mechanisms of traditional small-molecule chemotherapy-induced toxicities have been described, the absence of direct evidence warrants further research to identify the potential contribution of SLC transporters to the etiology of ADC-related toxicities.

## III Conclusions and future perspectives

The xenobiotic transporter field has greatly advanced in recent years. This has furthered our understanding of the function of SLC transporters and improved the translational and clinical application of this knowledge. Structural and computational advances have been made in the last decade with the advent of reliable cryo-electron microscopy technologies and more powerful data processing techniques.^493^^,^^494^ This has allowed for the generation of detailed structures of SLC transporters with substrates and inhibitors, thus improving our mechanistic understanding of their interactions.^495^ Improved screening technologies, both in silico and in vitro*,* offer accessible, unbiased, and reliable means to interrogate the transportome to an unprecedented degree.^39^^,^^43^ These computational and in vitro methodologies complement one another,^42^ especially in the context of large-scale genetic knock-out libraries.^39^ Leveraging CRISPR/Cas9 to identify the role of single SLC transporters in the cellular uptake of a drug will address fundamental questions regarding the predominant mechanisms by which drugs can cross membranes. The in vivo models that exist for the exploration of transporter-related phenomena, and the ease with which new models can be exploited, offer new pathways to demonstrate connections of SLC transporters to drug effects.^10^^,^^496^^,^^497^ Furthermore, large-scale omics data and the continuing investigation of novel pharmacogenomic and biomarker analyses are anticipated to contribute further to establishing the relevance of observations made preclinically to the clinic.^498^ Indeed, the sophistication of presently available models offers promising avenues for the translation of preclinical findings into safe and effective clinical interventions. As SLC transporter-mediated uptake becomes more widely recognized as an initiating event in chemotherapy-induced toxicity to healthy cells and organs, there is limitless potential for the improvement of the safety and efficacy of treatment regimens for patients.

In summary, there is extensive and accumulating evidence that SLC transporters are critical determinants of chemotherapy-related toxicity to normal tissue. However, our understanding of these phenomena remains incomplete, and our ability to predict or modulate them is imperfect. Chemotherapy-induced toxicities continue to be a detriment to successful cancer treatment, and an important contributing factor that degrades the quality of life of patients. The identification of SLC-dependent mechanisms in initiating these toxicities provides a unique opportunity for the development of therapeutic intervention strategies aimed at reducing local accumulation preceding the onset of injurious, intracellular signaling events. The widespread interrogation of the transportome in the context of chemotherapeutic agents and other common supportive care medications must be made a central priority to improve the care of cancer patients. Specifically, the rational design of polypharmacy treatments must consider interactions of xenobiotics with transporters so that (1) one drug does not unintentionally exacerbate the toxicity of another, and in fact (2) one drug can intentionally, or unintentionally, block the adverse tissue accumulation and subsequent injury of another. The development of this strategy is not only possible, given the field’s advances but necessary to ensure that we are offering completely optimized treatment regimens to patients and improving quality of life.

## Conflict of interest

No author has an actual or perceived conflict of interest with the contents of this article.
